# A schematic sampling protocol for contaminant monitoring in raptors

**DOI:** 10.1007/s13280-020-01341-9

**Published:** 2020-05-12

**Authors:** Silvia Espín, Jovan Andevski, Guy Duke, Igor Eulaers, Pilar Gómez-Ramírez, Gunnar Thor Hallgrimsson, Björn Helander, Dorte Herzke, Veerle L. B. Jaspers, Oliver Krone, Rui Lourenço, Pedro María-Mojica, Emma Martínez-López, Rafael Mateo, Paola Movalli, Pablo Sánchez-Virosta, Richard F. Shore, Christian Sonne, Nico W. van den Brink, Bert van Hattum, Al Vrezec, Chris Wernham, Antonio J. García-Fernández

**Affiliations:** 1 Area of Toxicology, Faculty of Veterinary Medicine, University of Murcia, Campus Espinardo, 30100 Murcia, Spain; 2 Vulture Conservation Foundation, Wuhrstrasse 12, 8003 Zurich, Switzerland; 3 Environmental Change Institute, Oxford University Centre for the Environment, South Parks Road, Oxford, OX1 3QY UK; 4 Department of Bioscience, Faculty of Technical Sciences, Aarhus University, Frederiksborgvej 399, POBox 358, 4000 Roskilde, Denmark; 5 Faculty of Life and Environmental Sciences, University of Iceland, Sturlugata 7, 102 Reykjavik, Iceland; 6 Environmental Research and Monitoring, Swedish Museum of Natural History, Frescativägen 40, PO Box 50007, 10405 Stockholm, Sweden; 7 NILU – Norwegian Institute for Air Research, Hjalmar Johansen Gate 14, 9296 Tromsö, Norway; 8 Environmental Toxicology Group, Department of Biology, Norwegian University of Science and Technology, Høgskoleringen 5, NO-7491 Trondheim, Norway; 9 Department of Wildlife Diseases, Leibniz Institut for Zoo and Wildlife Research, Alfred-Kowalke-Str. 17, 10315 Berlin, Germany; 10 MED - Mediterranean Institute for Agriculture, Environment and Development, LabOr, IIFA, Univ. Évora, Pólo da Mitra, Ap. 94, 7006-554 Évora, Portugal; 11 Santa Faz” Wildlife Rehabilitation Centre, Alicante, Generalitat Valenciana Spain; 12 Instituto de Investigación en Recursos Cinegéticos (IREC–CSIC, UCLMJCCM), Ronda de Toledo 12, 13005 Ciudad Real, Spain; 13 Naturalis Biodiversity Center, PO Box 9517, 2300 RA Leiden, The Netherlands; 14 UK Centre for Ecology & Hydrology, Lancaster Environment Centre, Library Avenue, Bailrigg, Lancaster, LA1 4AP UK; 15 Sub-Division of Toxicology, Wageningen University, Box 8000, 6700 EA Wageningen, The Netherlands; 16 Dep. Environment and Health, Faculty of Science, VU University Amsterdam, De Boelelaan 1085, 1081 HV Amsterdam, The Netherlands; 17 Department of Organisms and Ecosystems Research, National Institute of Biology, Večna pot 111, 1000 Ljubljana, Slovenia; 18 British Trust for Ornithology (Scotland), Unit 15 Beta Centre, Stirling University, Innovation Park, Stirling, FK9 4NF Scotland; 19 Slovenian Museum of Natural History, Prešernova 20, 1000 Ljubljana, Slovenia

**Keywords:** Best practices, Birds of prey, Falcons, Large-scale biomonitoring, Owls, Pan-European network

## Abstract

Birds of prey, owls and falcons are widely used as sentinel species in raptor biomonitoring programmes. A major current challenge is to facilitate large-scale biomonitoring by coordinating contaminant monitoring activities and by building capacity across countries. This requires sharing, dissemination and adoption of best practices addressed by the Networking Programme *Research and Monitoring for and with Raptors in Europe* (EURAPMON) and now being advanced by the ongoing international COST Action *European Raptor Biomonitoring Facility*. The present perspective introduces a schematic sampling protocol for contaminant monitoring in raptors. We provide guidance on sample collection with a view to increasing sampling capacity across countries, ensuring appropriate quality of samples and facilitating harmonization of procedures to maximize the reliability, comparability and interoperability of data. The here presented protocol can be used by professionals and volunteers as a standard guide to ensure harmonised sampling methods for contaminant monitoring in raptors.

**Electronic supplementary material:**

The online version of this article (10.1007/s13280-020-01341-9) contains supplementary material, which is available to authorized users.

## Introduction

Birds, and especially raptors (i.e. birds of prey, owls and falcons), have been widely used as sentinel species in biomonitoring programmes worldwide (Gómez-Ramírez et al. 2014; Espín et al. 2016). Such studies are used to evaluate spatiotemporal trends in contaminant concentrations and related effects and can provide early warning of emerging contaminant problems. In addition, they may be used to track the success of regulatory directives designed to protect humans, wildlife and the wider environment from pesticides and industrial contaminants (e.g. Council Regulation 315/93/EEC; REACH EU Regulation; Stockholm Convention on POPs; Aarhus Protocol on POPs; POPs regulation EU No. 2019/1021).

Because chemicals regulation is harmonised within the European Union, a major current challenge is to improve large-scale (pan-European) biomonitoring. This can be addressed by coordinating Europe-wide contaminant monitoring in raptors and by building capacity across countries. This requires sharing, dissemination and adoption of best practices. This was addressed by the Research Networking Programme *Research and Monitoring for and with Raptors in Europe* (EURAPMON, 2010–2015), funded by the European Science Foundation (https://www.eurapmon.net/) and is now being further advanced by the ongoing international COST Action, *European Raptor Biomonitoring Facility* (ERBFacility, CA16224, 2017–2021) (https://erbfacility.eu/). Under ERBFacility, three inter-linked scientific arenas are cooperating, the ‘analysis arena’ on ecotoxicological analyses, the ‘collections arena’ on storing and cataloguing raptor samples, and the ‘field arena’ on gathering additional samples and contextual field data (Duke et al. 2018; Movalli et al. 2019). This pan-European network of ornithologists, veterinary scientists, raptor ecologists, ecotoxicologists and analytical chemists will enable a new generation of research on environmental biomonitoring using raptors.

## Towards harmonisation and appropriate quality of samples for contaminant monitoring

In the EURAPMON programme it was identified that differences between studies in sampling and processing strategies for contaminant monitoring hampered direct comparison of study results and an integrated interpretation (Movalli et al. 2008). The use of appropriate sample containers and storage conditions was also identified as an essential step to avoid contamination of samples or degradation of the compound of interest, and information on this needed to be easily accessible to personnel collecting and sending samples to ecotoxicological laboratories. Although toxicology specialists have a deep understanding on these issues, other groups of professionals (e.g. field researchers) or volunteers (e.g. ringers) collecting samples need guidance that ensures appropriate collection of samples without compromising the analytical quality of the sample. Indeed, raptor population monitoring activities have the potential to enhance a widescale availability of raptor samples that are interlinked with key contextual data (e.g. breeding success, population trends, survival, diet, etc.), since some of the biological matrices needed for analysis (e.g. feathers, addled/deserted eggs, blood) are routinely collected as part of surveys of raptor breeding populations (Espín et al. 2016; Derlink et al. 2018). Consequently, it is essential to provide protocols on appropriate sampling methods for contaminant monitoring, for field ornithologists, personnel at museums and Wildlife Rehabilitation Centres, and other people involved in raptor sample collection.

A detailed best-practice sampling protocol was previously prepared by EURAPMON (Espín et al. 2014), as well as a publication of the potential widescale availability of raptor samples and the relative merits of each matrix type (Espín et al. 2016). However, through more recent meetings and workshops under ERBFacility involving researchers across 27 countries, the need to elaborate a protocol in a more schematic and clear format was identified. This schematic protocol should provide essential information on the practicalities of sampling and storage that were not earlier reported by Espín et al. 2014 (e.g. type of containers to conserve matrices, storage and transport conditions, and how these differ depending on the sample matrices and the contaminant to be analysed). Twenty-three researchers involved in the three arenas of the ERBFacility COST Action and representing 11 countries (Denmark, Germany, Iceland, Norway, Portugal, Slovenia, Spain, Sweden, Switzerland, The Netherlands and United Kingdom), have participated in the preparation of this schematic sampling protocol based upon their expertise in sampling and contaminant monitoring in raptors.

The protocol presented in this perspective (see Electronic Supplementary Material) provides guidance on sample collection for contaminant monitoring in raptors with a view to increasing sampling capacity across countries and facilitating harmonization of procedures to maximize the reliability, comparability and interoperability of biomonitoring data. While the protocol has been prepared under a Europe COST Action, the guidance is applicable to raptor monitoring worldwide. The protocol is presented as Supplementary Material to this communication, and has been prepared following an easy-to-follow style, with hyperlinks to redirect the reader to the relevant information elsewhere in the protocol.

**Fig. 1:**
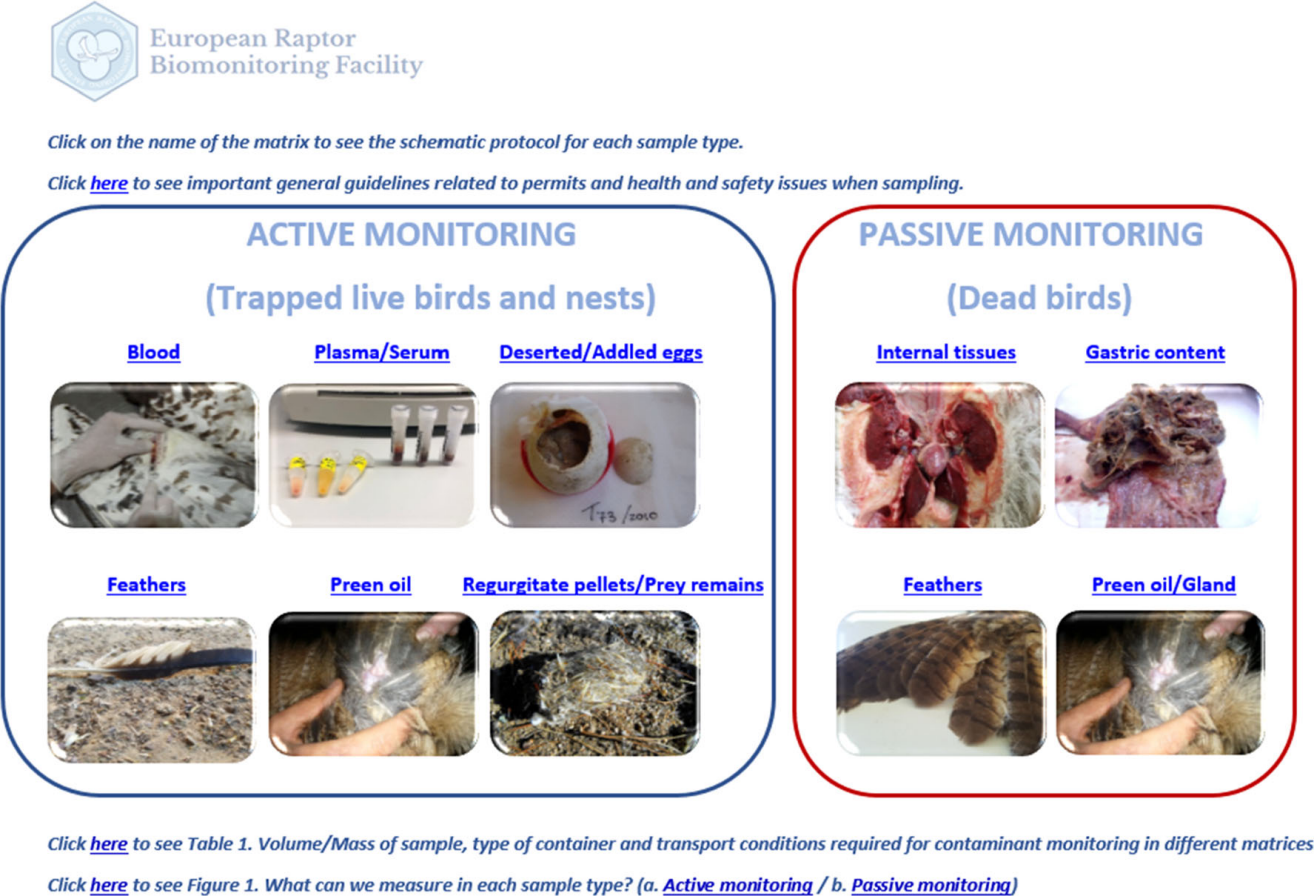
Preview of the main menu of the schematic protocol (full protocol presented as Supplementary Material)

**Fig. 2:**
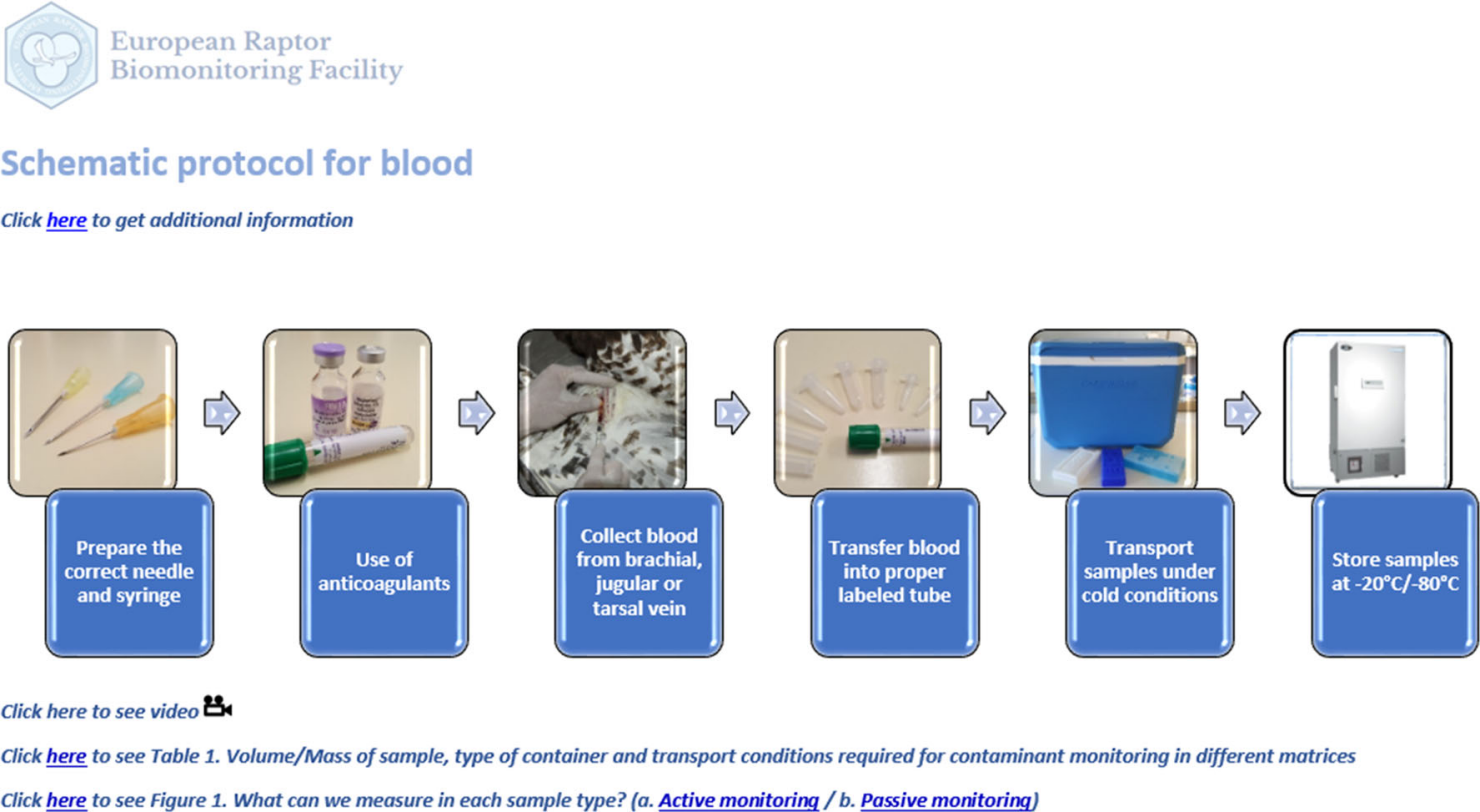
Preview of the schematic protocol for blood sampling (full protocol presented as Supplementary Material)

The schematic protocol starts with each matrix type, including sample types that are collected both during active sampling (samples taken from captured live birds and monitored nests) and passive monitoring (samples taken from dead birds and deserted nests) (Fig. 1). These sample types include whole blood, plasma, serum, deserted or addled eggs, feathers, preen oil, regurgitated pellets, prey remains, gastric content and internal tissues. The reader clicks on the sample type of interest and is redirected to the specific protocol for each matrix (e.g. see protocol for blood sampling in Fig. 2), which in turn offers further hyperlinks to additional and more detailed information. Some important general guidelines are also given regarding sampling and ethical permits, personal safety and wildlife health, animal welfare, labelling samples, and essential guidance to avoid contamination and to record basic data (date, location, etc.) in the sampling report. Moreover, new information is provided on the volume/mass of sample needed for contaminant monitoring, the most suitable container type to conserve the sample, and the necessary conditions required for transportation and storage.

Different sample matrices provide different information about exposure and effects and not all are suitable for biomonitoring (Espín et al. 2016). Thus, information on the type of contaminants and biomarkers most frequently analysed in the different matrices in live and dead birds is also shown. Photographs and links to web-based videos are also provided to illustrate the proper materials and methods needed for sample collection, taking measurements (such as measuring egg size, eggshell thickness, body fat tissue during necropsy, etc.) and identifying gonads and internal tissues.

We recommend the schematic protocol for use by professionals and volunteers as a standard guide to ensure harmonised sampling methods and appropriate quality of samples collected for contaminant monitoring in raptors. Contaminant issues often occur across national and continental boundaries, and therefore require harmonised methods to study their occurrence, impact and any effect of legal or voluntary mitigation, whether of legacy, current or emerging contaminants.

## Electronic supplementary material

Below is the link to the electronic supplementary material.

(PDF 3650 kb)
