# Supplementary material for: A schematic sampling protocol for contaminant monitoring in raptors
Source: Ambio. 2020 May 12;50(1):95–100. doi: 10.1007/s13280-020-01341-9 (PMC7708607; doi:10.1007/s13280-020-01341-9)
Supplement: Supplementary file 1 — (PDF 3650 kb) [file 13280_2020_1341_MOESM1_ESM.pdf]

Ambio

Electronic Supplementary Material

This supplementary material has not been peer reviewed

Title: **A schematic sampling protocol for contaminant monitoring in raptors**

Authors: Silvia Espín, Jovan Andevski, Guy Duke, Igor Eulaers, Pilar Gómez-Ramírez, Gunnar Thor Hallgrimsson, Björn Helander, Dorte Herzke, Veerle L. B. Jaspers, Oliver Krone, Rui Lourenço, Pedro María-Mojica, Emma Martínez-López, Rafael Mateo, Paola Movalli, Pablo Sánchez-Virosta, Richard F. Shore, Christian Sonne, Nico W. van den Brink, Bert van Hattum, Al Vrezec, Chris Wernham, Antonio J. García-Fernández

European Raptor
Biomonitoring Facility

A schematic sampling protocol for contaminant monitoring in raptors

Silvia Espín[1]*, Jovan Andevski[2], Guy Duke[3], Igor Eulaers[4], Pilar Gómez-Ramírez[1], Gunnar Thor Hallgrimsson[5], Björn Helander[6], Dorte Herzke[7], Veerle L. B. Jaspers[8], Oliver Krone[9], Rui Lourenço[10], Pedro María-Mojica[1,11], Emma Martínez-López[1], Rafael Mateo[12], Paola Movalli[13], Pablo Sánchez-Virosta[1], Richard F. Shore[14], Christian Sonne[4], Nico W. van den Brink[15], Bert van Hattum[16], Al Vrezec[17], Chris Wernham[18], Antonio J. García-Fernández[1]

[1]Area of Toxicology, Faculty of Veterinary Medicine, University of Murcia, Campus Espinardo, 30100 Murcia, Spain
[2]Vulture Conservation Foundation, Wuhrstrasse 12, CH-8003 Zurich, Switzerland
[3]Environmental Change Institute, Oxford University Centre for the Environment, South Parks Road, Oxford, OX1 3QY, UK
[4]Department of Bioscience, Faculty of Technical Sciences, Aarhus University, DK-4000 Roskilde, Denmark
[5]Faculty of Life and Environmental Sciences, University of Iceland, Sturlugata 7, 102 Reykjavik, Iceland
[6]Environmental Research and Monitoring, Swedish Museum of Natural History, Frescativägen 40, PO Box 50007, SE-104 05 Stockholm, Sweden
[7]NILU – Norwegian Institute for Air Research, 9296 Tromsø, Norway
[8]Environmental Toxicology Group, Department of Biology, Norwegian University of Science and Technology, Høgskoleringen 5, NO-7491, Trondheim, Norway
[9]Department of Wildlife Diseases, Leibniz Institut for Zoo and Wildlife Research Alfred-Kowalke-Str. 17, 10315 Berlin, Germany
[10]MED - Mediterranean Institute for Agriculture, Environment and Development, LabOr, IIFA, Univ. Évora, Pólo da Mitra, Ap. 94, 7006-554, Évora, Portugal
[11]"Santa Faz" Wildlife Rehabilitation Centre, Alicante, Generalitat Valenciana, Spain
[12]Instituto de Investigación en Recursos Cinegéticos (IREC–CSIC, UCLM, JCCM), Ronda de Toledo 12, 13005, Ciudad Real, Spain
[13]Naturalis Biodiversity Center, PO Box 9517, 2300 RA Leiden, The Netherlands
[14]UK Centre for Ecology & Hydrology, Lancaster Environment Centre, Library Avenue, Bailrigg, Lancaster LA1 4AP, UK
[15]Sub-division of Toxicology, Wageningen University, Box 8000 6700 EA, Wageningen, The Netherlands
[16]Dep. Environment and Health, Faculty of Science, VU University Amsterdam, De Boelelaan 1085, 1081 HV Amsterdam, The Netherlands
[17]Department of Organisms and Ecosystems, National Institute of Biology, Večna pot 111, 1000 Ljubljana, Slovenia
[18]British Trust for Ornithology (Scotland), Unit 15 Beta Centre, Stirling University Innovation Park, Stirling, FK9 4NF, Scotland

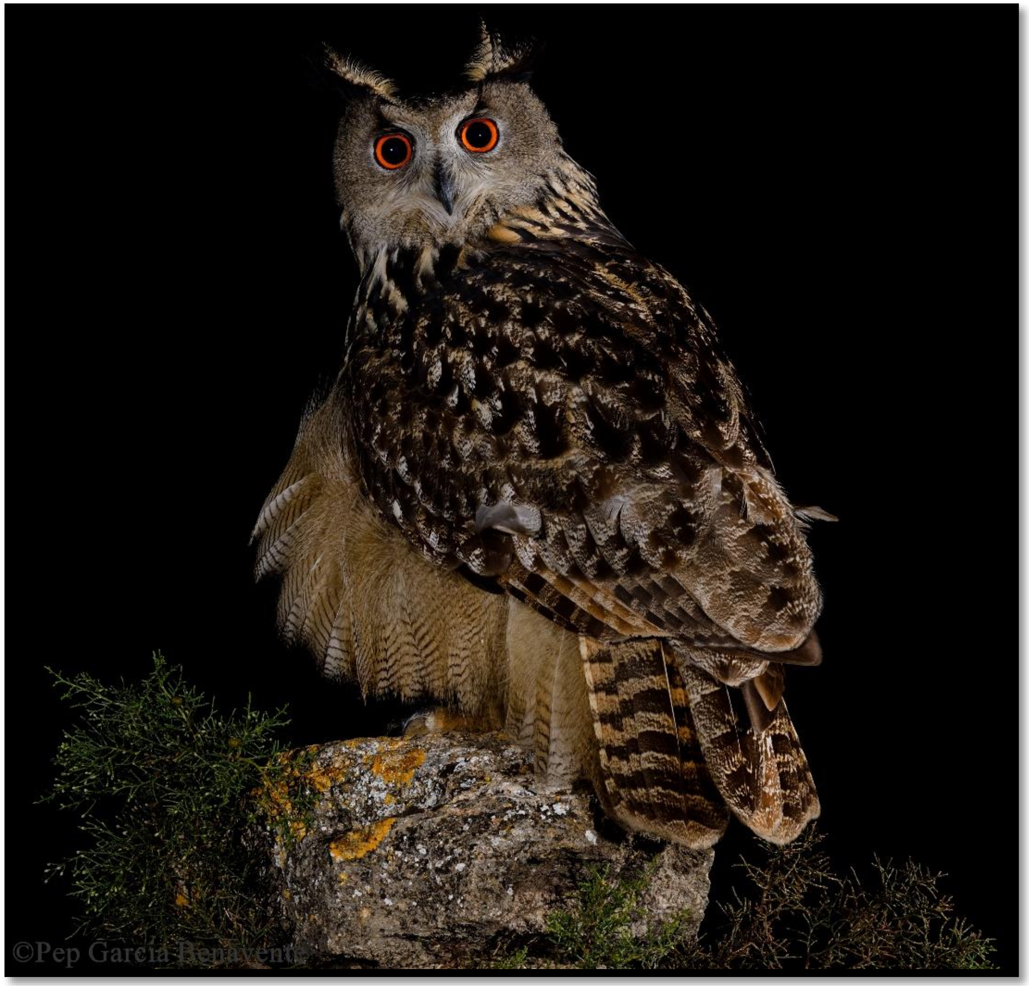

*Corresponding author: silvia.espin@um.es / Tel. 0034868884317
Area of Toxicology, Faculty of Veterinary Medicine, University of Murcia, Campus Espinardo, 30100 Murcia, Spain

Cover picture: José García Benavente

TABLE OF CONTENTS

Schematic protocol - Main menu	3
Important general guidelines	4
Schematic protocol for blood	5
Schematic protocol for plasma / serum	6
Schematic protocol for deserted and addled eggs	7
Schematic protocol for feathers	8
Schematic protocol for preen oil	9
Schematic protocol for regurgitate pellets / prey remains	10
Schematic protocol for internal tissues / gastric content	11
Schematic protocol for blood / plasma / serum / red blood cells: additional information	16
Schematic protocol for deserted and addled eggs: additional information	18
Schematic protocol for feathers: additional information	20
Schematic protocol for preen oil: additional information	21
Schematic protocol for regurgitate pellets / prey remains: additional information	21
Schematic protocol for internal tissues / gastric content: additional information	22
References	27

Click on the name of the matrix to see the schematic protocol for each sample type.

Click here to see important general guidelines related to permits and health and safety issues when sampling.

ACTIVE MONITORING

(Trapped live birds and nests)

Blood

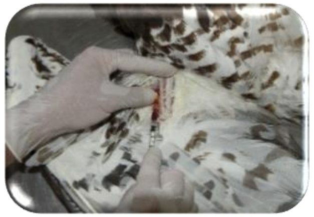

Plasma/Serum

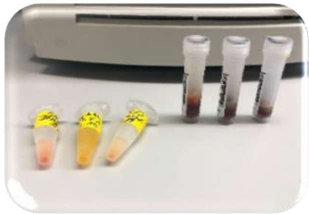

Deserted/Addled eggs

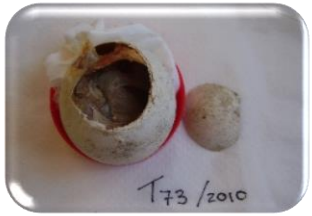

Feathers

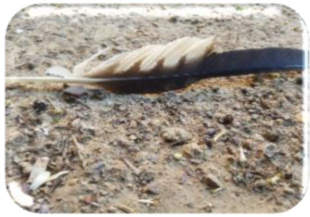

Preen oil

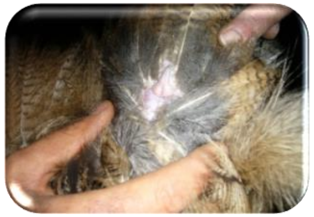

Regurgitate pellets/Prey remains

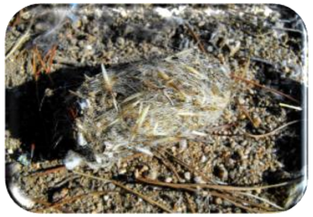

PASSIVE MONITORING

(Dead birds)

Internal tissues

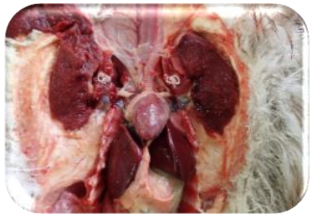

Gastric content

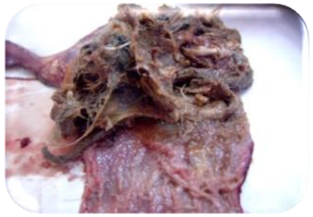

Feathers

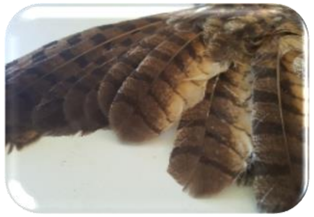

Preen oil/Gland

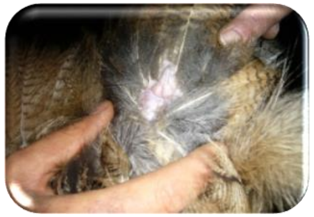

Click here to see Table 1. Volume/Mass of sample, type of container and transport conditions required for contaminant monitoring in different matrices

Click here to see Figure 1. What can we measure in each sample type? (a. Active monitoring / b. Passive monitoring)

Important general guidelines

These guidelines do not include any destructive sampling.
Permits: Approach and disturbance of nest sites and handling/sampling of protected species is regulated in most countries. All necessary licenses and permissions must be obtained from the appropriate national agencies before work is undertaken (permission to visit active nests, permission for handling and taking samples, CITES or other legislation for holding/storing samples, accreditation of personnel). Samples should be collected by trained and authorised personnel.
Personal safety and wildlife health: Use appropriate personal protective equipment to avoid zoonotic diseases or zoonoses. Put an effective barrier between you and the disease agent, such as masks, gloves, coveralls, boots, goggles, and respirators. The level of personal protection required depends on the situation. Prevent mechanical transmission of disease agents through disinfecting supplies and equipment between sites and/or animals. Apply safety requirements for climbing and hiking. Do not go into the field alone, risk assess the situation in advance, and have mobile or satellite phones (depending on the location) plus appropriate contact numbers.
Animal welfare: Avoid posing unnecessary stress to the birds. Cover the head with a cloth, avoid unnecessary noise and talking. In case of sampling of living animals, avoid periods of the day with extreme temperatures (or extreme rain) in order to limit heat stress. Keep the handling time as short as possible. Avoid multiple handling of a bird. If possible, do sampling at same time as ringing to limit disturbance and stress to the bird. Leave the nest site as you found it.
Sample amount: Check with the laboratory for minimal requirements (see Table 1), but in case of invasive sampling (blood, plucked feathers) take no more sample than safe for the bird (see details on the different sampling protocols).
Identification: Always note information systematically following a previously prepared scheme regarding the characteristics and circumstances of the samples collected. Label the individual sample containers prior or immediately after the sample is collected. Each sample should be identifiable from a unique code. A short and self-explanatory identification system that is easy to implement in the field should be used.
Avoid contamination: Use appropriate material for sampling and storage conditions according to the aims of the study (take advice from the laboratory undertaking the chemical analysis; see Table 1). Handle the containers carefully to avoid contamination. Containers may be specifically precleaned for some contaminants (e.g. POPs, metals, perfluorinated; ask the laboratory). Keep some sampling material/containers for blank/control analyses. Do not smoke, drink or eat during sampling. Do not wear perfume/deodorant (as personal care products are now turning up as emerging contaminants).
Basic data and records: The following information should be clearly described in the sampling report, which must always accompany the samples submitted for analysis: Date and time of sampling, study area (country, province, and specific location - there may be a need to keep specific location data confidential to avoid disturbance, illegal persecution or illegal egg and specimen collection; if not specify precise latitude and longitude to nearest second to be recorded, to aid subsequent analysis/interpretation), type of samples and number of samples collected, biological data (species scientific name and common name, ringing data if possible, age and gender if can be determined, morphometric measurements e.g. weight, head and bill length, wing length, tarsus length, and tarsus width, body condition index, nest information e.g. numbers of eggs, live and dead nestlings, unhatched eggs, eggshells), and other general observations (e.g. prey remains in the nest, birds in bad conditions, etc.).

See References here
Go back to the main menu here

European Raptor
Biomonitoring Facility

Schematic protocol for blood

Click [here](#) to get additional information

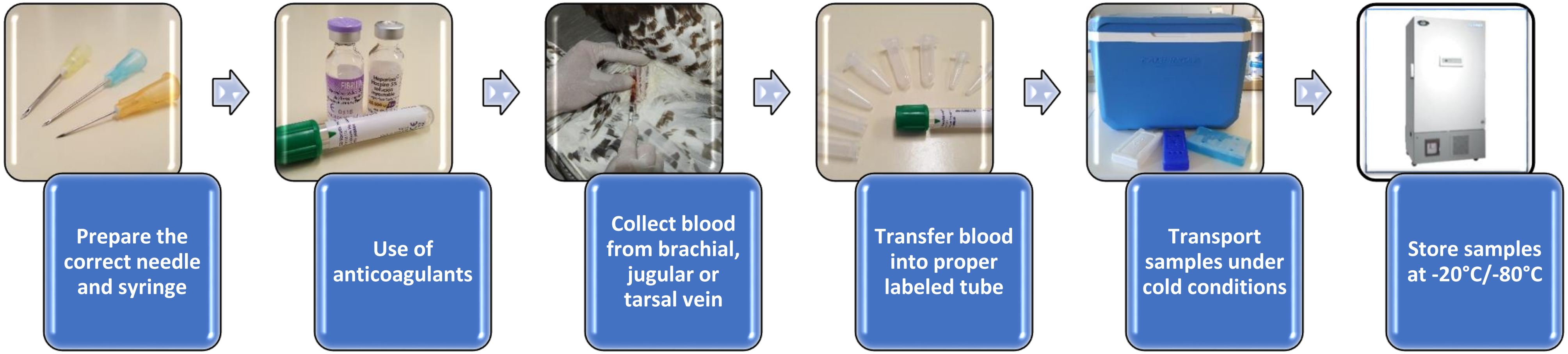

Click [here](#) to see video

Click [here](#) to see Table 1. Volume/Mass of sample, type of container and transport conditions required for contaminant monitoring in different matrices

Click [here](#) to see Figure 1. What can we measure in each sample type? (a. [Active monitoring](#) / b. [Passive monitoring](#))

Go back to the main menu [here](#)

Schematic protocol for plasma / serum

Click here to get additional information

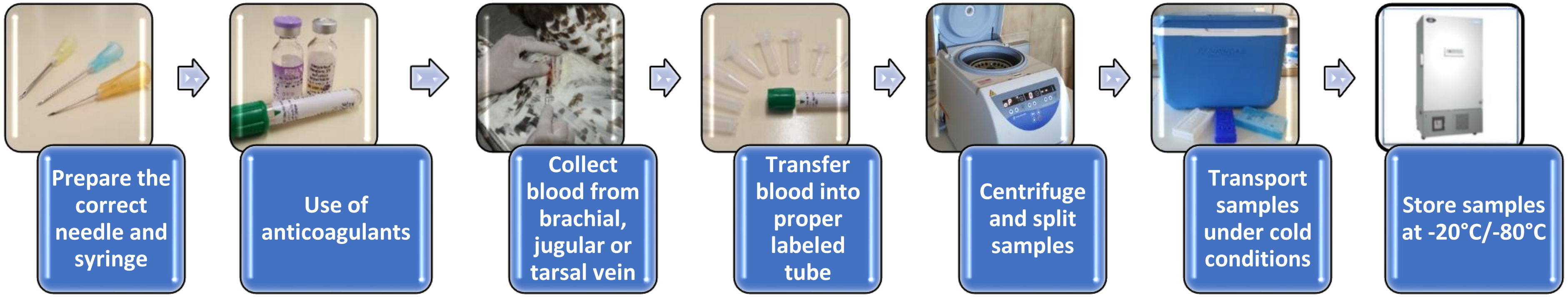

Click to see video for blood extraction and plasma/serum separation

Click here to see Table 1. Volume/Mass of sample, type of container and transport conditions required for contaminant monitoring in different matrices

Click here to see Figure 1. What can we measure in each sample type? (a. Active monitoring / b. Passive monitoring)

Go back to the main menu here

Schematic protocol for deserted and addled eggs

Click <u>here</u> to get additional information

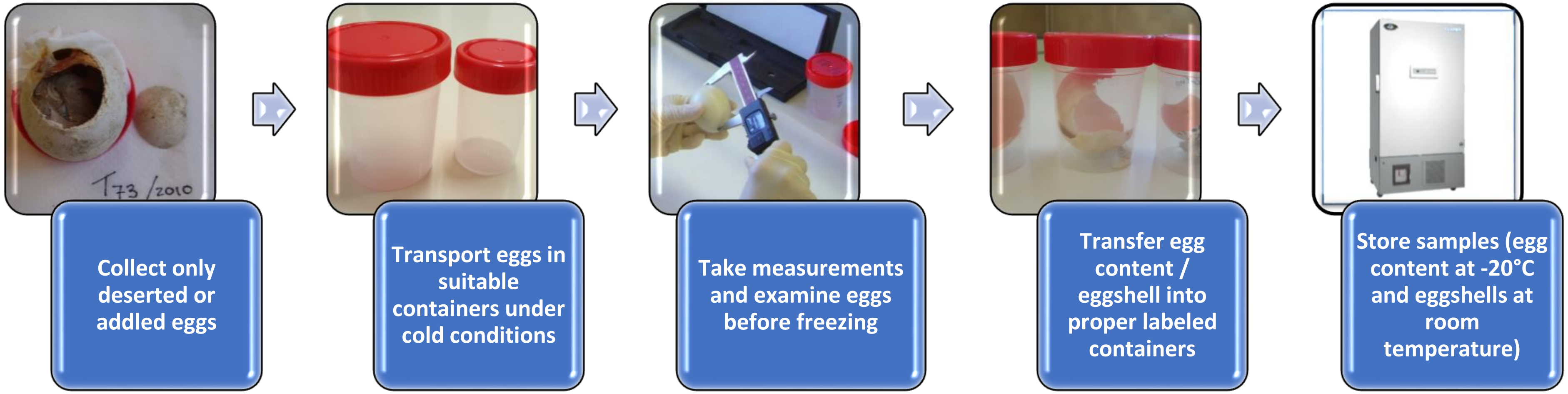

Click <u>here</u> to see video

Click <u>here</u> to see Table 1. Volume/Mass of sample, type of container and transport conditions required for contaminant monitoring in different matrices

Click <u>here</u> to see Figure 1. What can we measure in each sample type? (a. <u>Active monitoring</u> / b. <u>Passive monitoring</u>)

Go back to the main menu <u>here</u>

Schematic protocol for feathers

Click [here](#) to get additional information

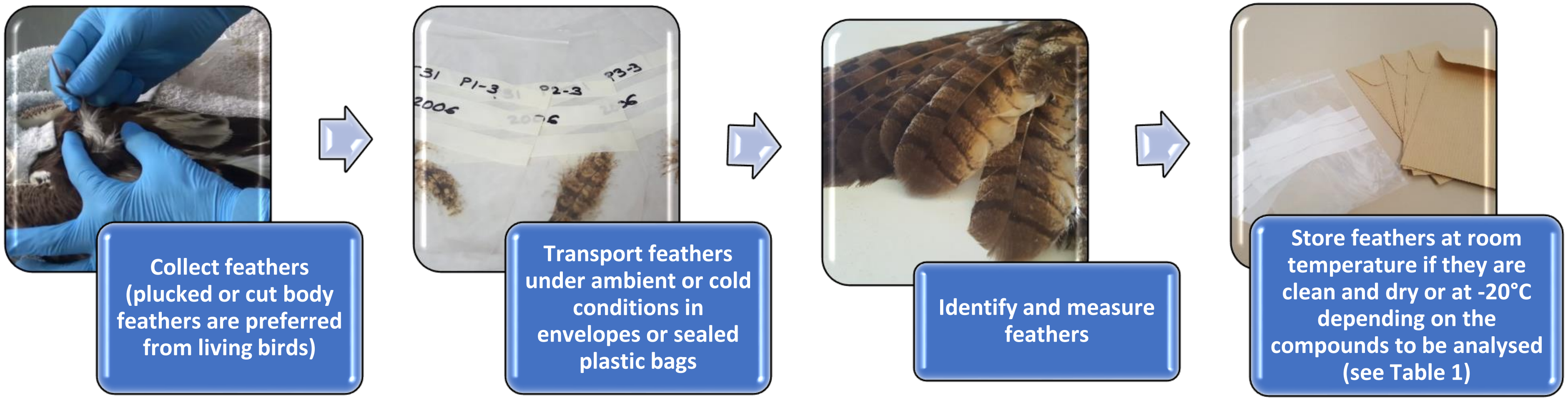

Click [here](#) to see video

Click [here](#) to see Table 1. Volume/Mass of sample, type of container and transport conditions required for contaminant monitoring in different matrices

Click [here](#) to see Figure 1. What can we measure in each sample type? (a. [Active monitoring](#) / b. [Passive monitoring](#))

Go back to the main menu [here](#)

Schematic protocol for preen oil

Click here to get additional information

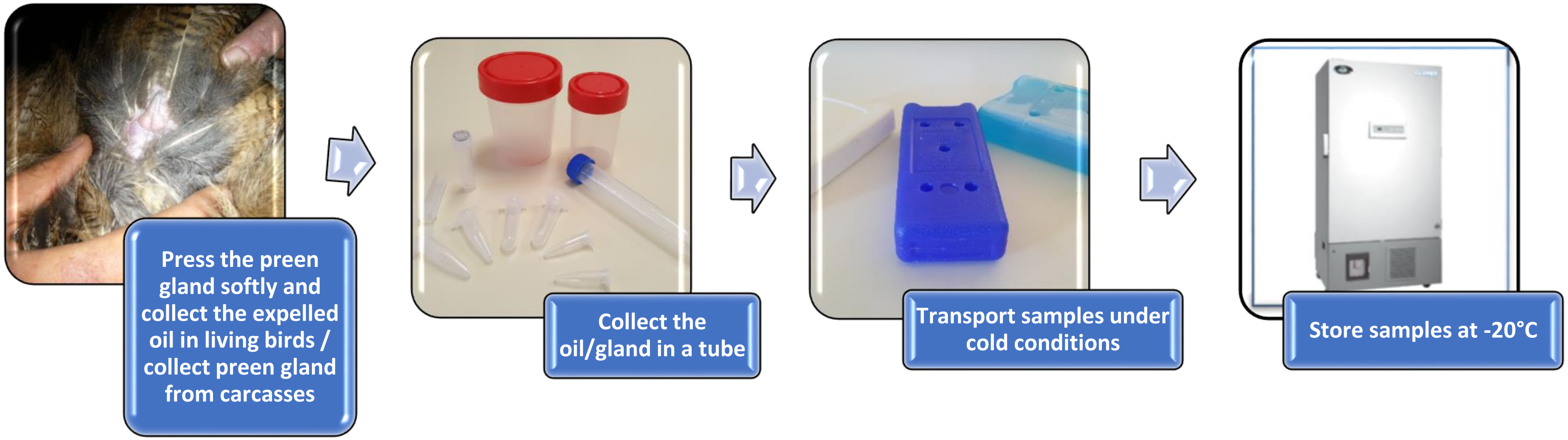

Click here to see Table 1. Volume/Mass of sample, type of container and transport conditions required for contaminant monitoring in different matrices

Click here to see Figure 1. What can we measure in each sample type? (a. Active monitoring / b. Passive monitoring)

Go back to the main menu here

Schematic protocol for regurgitate pellets/prey remains

Click here to get additional information

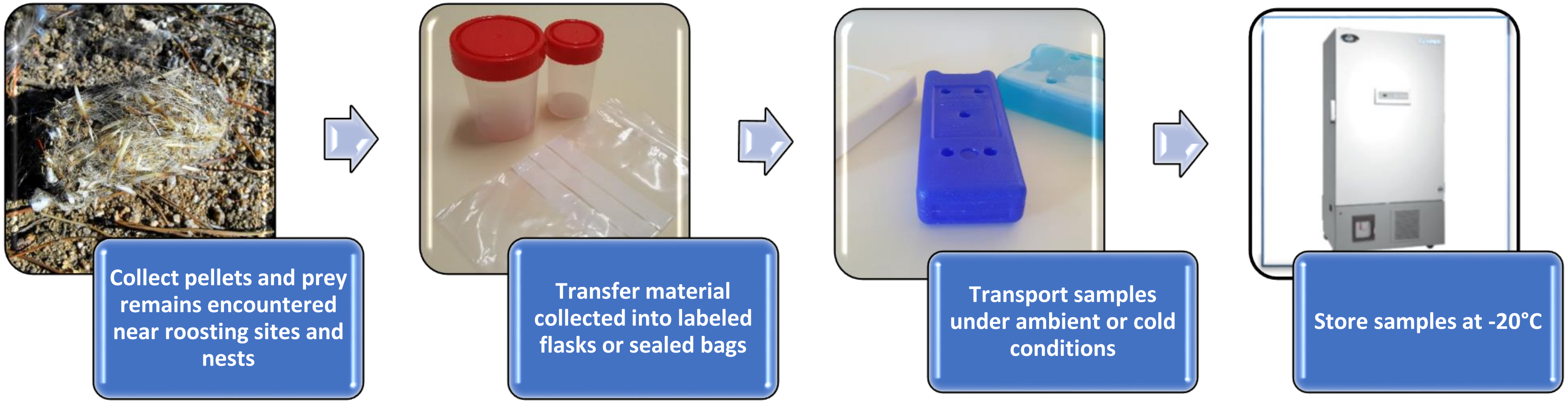

Click here to see Table 1. Volume/Mass of sample, type of container and transport conditions required for contaminant monitoring in different matrices

Click here to see Figure 1. What can we measure in each sample type? (a. Active monitoring / b. Passive monitoring)

Go back to the main menu here

Schematic protocol for internal tissues/gastric content

Click here to get additional information

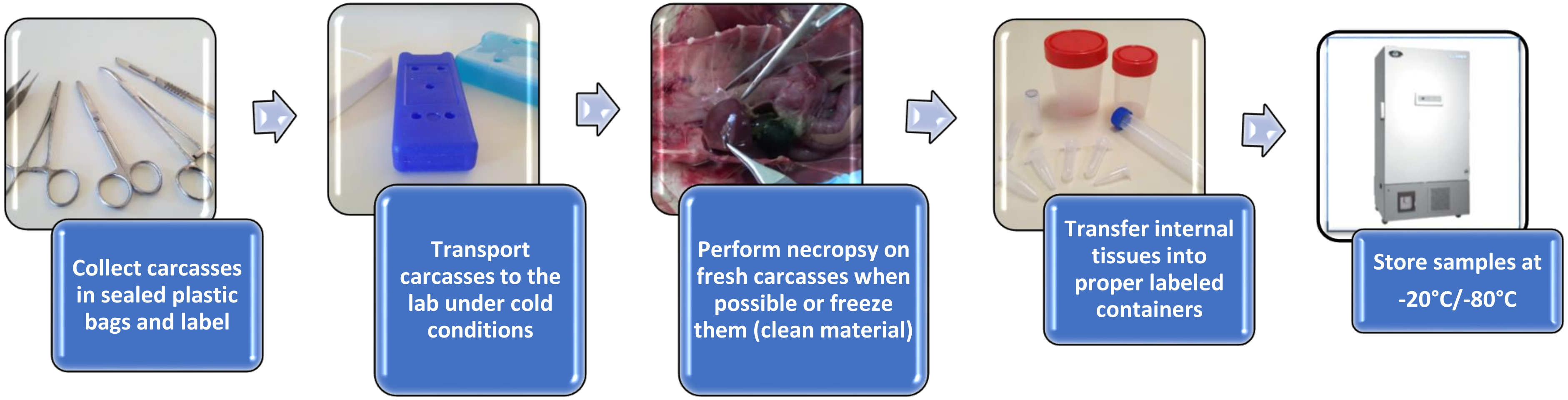

Click here to see video

Click here to see Table 1. Volume/Mass of sample, type of container and transport conditions required for contaminant monitoring in different matrices

Click here to see Figure 1. What can we measure in each sample type? (a. Active monitoring / b. Passive monitoring)

Go back to the main menu here

Table 1. Volume/Mass of sample, type of container and transport conditions required for contaminant monitoring in different matrices

		Matrix[a]	Blood[b]	Plasma/serum[b]	Feathers[c]	Eggs[d]	Liver[e]	Kidney[e]	Brain[e]	Bone[e]	Muscle[e]	Fat[e]	Preen oil	Regurgitated pellets / Prey remains
**Metals (Pb/Hg)**	Volume/Mass (range)		0.1-0.25 ml (~1 ml when using AAS)	NA	ca. 0.1-0.2 g (min. 0.02 g when using DMA for Hg and 0.1 g when using ICP-MS); BF: 5-10 units; TF/WF: 1-2 units	0.2-0.5 g ww (min. 0.02 g when using DMA for Hg, 0.1 g when using ICP-MS, ~3g when using AAS); whole egg if	0.2-0.5 g ww (min. 0.02 g when using DMA for Hg, 0.1 g when using ICP-MS, ~3g when using AAS)	0.2-0.5 g ww (min. 0.02 g when using DMA for Hg, 0.1 g when using ICP-MS, ~3g when using AAS)	0.2-0.5 g ww (min. 0.02 g when using DMA for Hg, 0.1 g when using ICP-MS, ~3g when using AAS)	0.2-0.5 g ww (~3g when using AAS)	0.2-0.5 g ww	0.2-0.5 g ww (min. 0.02 g when using DMA for Hg, 0.1 g when using ICP-MS, ~3g when using AAS)	MI	What you find in the field
	Type of container		PP tubes (metal free)	NA	Sealed plastic bag / Envelope	PP jar (metal free)	PP jar (metal free)	PP jar (metal free)	PP jar (metal free)	PP jar (metal free)	PP jar (metal free)	PP jar (metal free)	MI	Sealed plastic bag
	Transport conditions	Temperature	Cold blocks	NA	Ambient temperature/Cold blocks	Cold blocks	Cold blocks	Cold blocks	Cold blocks	Cold blocks	Cold blocks	Cold blocks	MI	Ambient temperature / Cold blocks
		Time	ca. 24 h	NA	Indef	ca. 24 h	ca. 24 h	ca. 24 h	ca. 24 h	ca. 24 h	ca. 24 h	ca. 24 h	MI	Indef (ca. 24 h for prey remains)
	Storage conditions	Temperature	−20°C	NA	Ambient temperature/ −20°C [1] (preferrably in darkness)	−20°C	−20°C	−20°C	−20°C	−20°C	−20°C	−20°C	MI	Ambient temperature / −20°C (prey remains)
		Time	Indef	NA	Indef	Indef	Indef	Indef	Indef	Indef	Indef	Indef	MI	Indef
**Agrochemicals**	Volume/Mass (range)		1-2 ml	0.2 ml	0.2-0.5 g; BF: 5-10 units; TF/WF: 1-2 units	0.2-3 g; whole egg	0.2-3 g	0.2-3 g	0.2-3 g	NA	0.2-3 g	0.2-3 g	0.01-0.1 g	For prey remains different tissues could be analysed (see other columns)
	Type of container		PP tubes	PP tubes	Plastic sealed bag / Envelope	PP jar	PP jar	PP jar	PP jar	NA	PP jar	PP jar	PP jar	
	Transport conditions	Temperature	Cold blocks	Cold blocks	Ambient temperature / Cold blocks	Cold blocks	Cold blocks	Cold blocks	Cold blocks	NA	Cold blocks	Cold blocks	Cold blocks	
		Time	ca. 24 h	ca. 24 h	Indef	ca. 24 h	ca. 24 h	ca. 24 h	ca. 24 h	NA	ca. 24 h	ca. 24 h	ca. 24 h	
	Storage conditions	Temperature	−20°C	−20°C	Ambient temperature/ −20°C [1] (preferrably in darkness)	−20°C	−20°C	−20°C	−20°C	NA	−20°C	−20°C	−20°C	
		Time	See note [4]	See note [4]	See note [4]	See note [4]	See note [4]	See note [4]	See note [4]	NA	See note [4]	See note [4]	See note [4]	

Abbreviations and notes can be found under the table (next page)

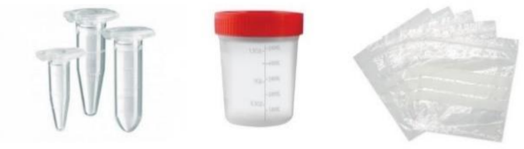

Example of containers: polypropylene (PP) tubes, PP jar and sealed (to avoid freezer burn) plastic bags. Although PP containers are recommended in general, glass could be considered if practical (note that some compounds varying from flame retardants to plasticisers could be in plastics and there could be potential contamination, so take advice from the laboratory and use field blanks when possible). Containers may be specifically precleaned for some contaminants (e.g. POPs, metals, perfluorinated; ask the laboratory).

Go back to the main menu here

Table 1. Volume/Mass of sample, type of container and transport conditions required for contaminant monitoring in different matrices

Matrix[a]			Blood[b]	Plasma/serum[b]	Feathers[c]	Eggs[d]	Liver[e]	Kidney[e]	Brain[e]	Bone[e]	Muscle[e]	Fat[e]	Preen oil	Regurgitated pellets / Prey remains
**Pharmaceuticals**	Volume/Mass (range)		P-NC	0.1-0.25 ml	1 g	2 g	2 g	2 g	2 g	NA	2 g	2 g	MI	For prey remains different tissues could be analysed (see other columns)
	Type of container		P-NC	PP tubes [2]	Sealed plastic bag / Envelope [2]	PP jar [2]	PP jar [2]	PP jar [2]	PP jar [2]	NA	PP jar [2]	PP jar [2]	MI	
	Transport conditions	Temperature	P-NC	Cold blocks	Cold blocks	Cold blocks	Cold blocks	Cold blocks	Cold blocks	NA	Cold blocks	Cold blocks	MI	
		Time	P-NC	< 24 h	< 24 h	< 24 h	< 24 h	< 24 h	< 24 h	NA	< 24 h	< 24 h	MI	
	Storage conditions	Temperature	P-NC	−20°C/−80°C [3]	−20°C/−80°C [2] (preferrably in darkness)	−20°C	−20°C	−20°C	−20°C	NA	−20°C	−20°C	MI	
		Time	P-NC	See note [4]	See note [4]	See note [4]	See note [4]	See note [4]	See note [4]	NA	See note [4]	See note [4]	MI	
**Rodenticides**	Volume/Mass (range)		1 ml	1 ml	NA	0.5-2 g	0.5-2 g	MI	NA	NA	MI	NA	NA	Plastic sealed bag for pellets. For prey remains different tissues could be analysed (see other columns)
	Type of container		PP tubes	PP tubes	NA	PP jar	PP jar	MI	NA	NA	MI	NA	NA	
	Transport conditions	Temperature	Cold blocks	Cold blocks	NA	Cold blocks	Cold blocks	MI	NA	NA	MI	NA	NA	
		Time	< 24 h	< 24 h	NA	< 24 h	< 24 h	MI	NA	NA	MI	NA	NA	
	Storage conditions	Temperature	−20°C	−20°C	NA	−20°C	−20°C	MI	NA	NA	MI	NA	NA	
		Time	See note [5]	See note [5]	NA	See note [5]	See note [5]	MI	NA	NA	MI	NA	NA	
**Perfluorinated compounds**	Volume/Mass (range)		0.2-1 ml	min. 0.2 ml	ca. 0.1-1 g	0.5-1 g	ca. 1 g	ca. 1 g	ca. 1 g	MI	ca. 1 g	0.5-1 g	0.01-0.1 g	ca. 1 g
	Type of container		PP tubes	PP tubes	Sealed plastic bag / Envelope	PP jar	PP jar	PP jar	PP jar	MI	PP jar	PP jar	PP jar	PP jar
	Transport conditions	Temperature	Cold blocks (<4°C)	Cold blocks	Ambient temperature/Cold blocks	Cold blocks	Cold blocks	Cold blocks	Cold blocks	MI	Cold blocks	Cold blocks	Cold blocks	Cold blocks
		Time	ca. 24 h	ca. 24 h	ca. 24 h	ca. 24 h	ca. 24 h	ca. 24 h	ca. 24 h	MI	ca. 24 h	ca. 24 h	ca. 24 h	ca. 24 h
	Storage conditions	Temperature	−20°C	−20°C	Ambient temperature/ −20°C [1] (preferrably in darkness)	−20°C	−20°C	−20°C	−20°C	MI	−20°C	−20°C	−20°C	−20°C
		Time	Indef	Indef	Indef	Indef	Indef	Indef	Indef	MI	Indef	Indef	Indef	Indef

[a] Please note that these are general guidelines. Take advice from the laboratory undertaking the chemical analysis.
[b] Volume criteria: A general rule is that the collection weight should not exceed 2% of the body weight of the animal in any 14-day period, or 1% at any one time. Values provided in the table are volume/mass ranges generally needed in the Toxicology lab for analysis, but it will depend on the technique used.
[c] From live birds, plucked contour body feathers (e.g. back/breast feathers) are preferred. Moulted feathers, chick down feathers and feathers from museum specimens are also useful. Consideration should be given to possible external contamination of museum feathers, e.g. due to conservation treatments.
[d] This protocol does only deal with non-destructive sampling, thus it only refers to deserted or addled eggs.
[e] Internal tissues collected during necropsies.
[1] For feathers when they are wet or have tissue/blood attached to them, they need to be cleaned/dried or they need to be stored in a freezer and not room temperature (as then this may lead to further decay)
[2] Pharmaceuticals is a broad group, and plastic containers may contain some compounds (e.g. UV filters), this should be considered or part of the plastic container analysed. Take advice from the laboratory undertaking the analysis.
[3] −80°C recommended for some drugs and for long storage periods (> 3 months), take advice from the laboratory undertaking the analysis.
[4] Agrochemicals and pharmaceuticals are broad groups. Some are not easy to break down (e.g. PCBs and most chlorinated pesticides has been found to be stable for at least one year at −20°C) but others may be rapidly degraded over time (e.g. significant degradation of some antibiotics after 2-24 weeks depending on the compound and tissue type when conserved at −20°C; O'Brien et al. 1981; Vanderkop et al. 1989; or metamidophos insecticide after 60-90 days in liver samples conserved at −20°C; MacLachlan et al. 2003). −80°C would be to recommend for long-time banking of soft tissues.
[5] Few studies have been done investigating the stability of rodenticides in frozen samples (eg. bromadiolone concentrations decreased 6-41% in whole blood samples stored at −20°C after 83-201 days; Vindenes et al., 2008). −80°C would be to recommend for long-time banking of soft tissues.
BF:Body feathers, TF/WF:Tail feathers/Wing feathers, ww: wet weight, PP:Polypropylene, Indef: Indefinitely (consider dessication), NA:Not applicable (sample type not useful for that group of compounds), MI: more information is needed, P-NC: possible but not the sample of choice

See References here
Go back to the main menu here

Figure 1a. What can we measure in each sample type? – Active monitoring

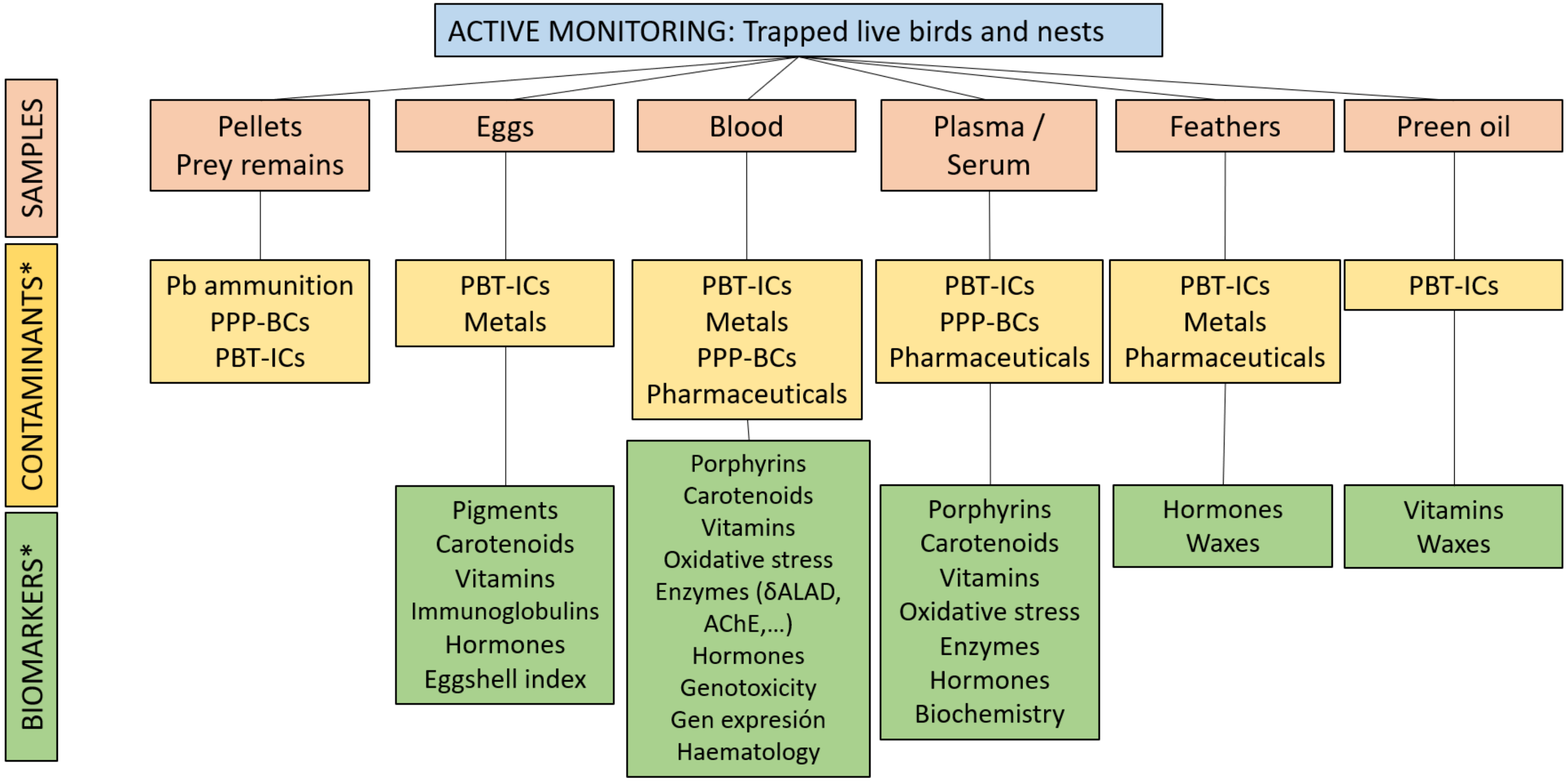

PBT-ICs: persistent bioaccumulative toxicants (chlorinated, brominated) + industrial compounds (fluorinated, PAHs,…)
PPP-BCs: plant protection products and biocides (insecticides, rodenticides, fungicides, herbicides,…)
δALAD: delta aminolevulinic acid deshidratase / **AChE**: acetylcholinesterase
*Contaminants and biomarkers frequently analyzed in biomonitoring studies are shown. Please see Espín et al. (2016) to see specific merits of each type of sample for contaminant analysis.

See References here
Go back to the main menu here

Figure 1b. What can we measure in each sample type? -**Passive monitoring**

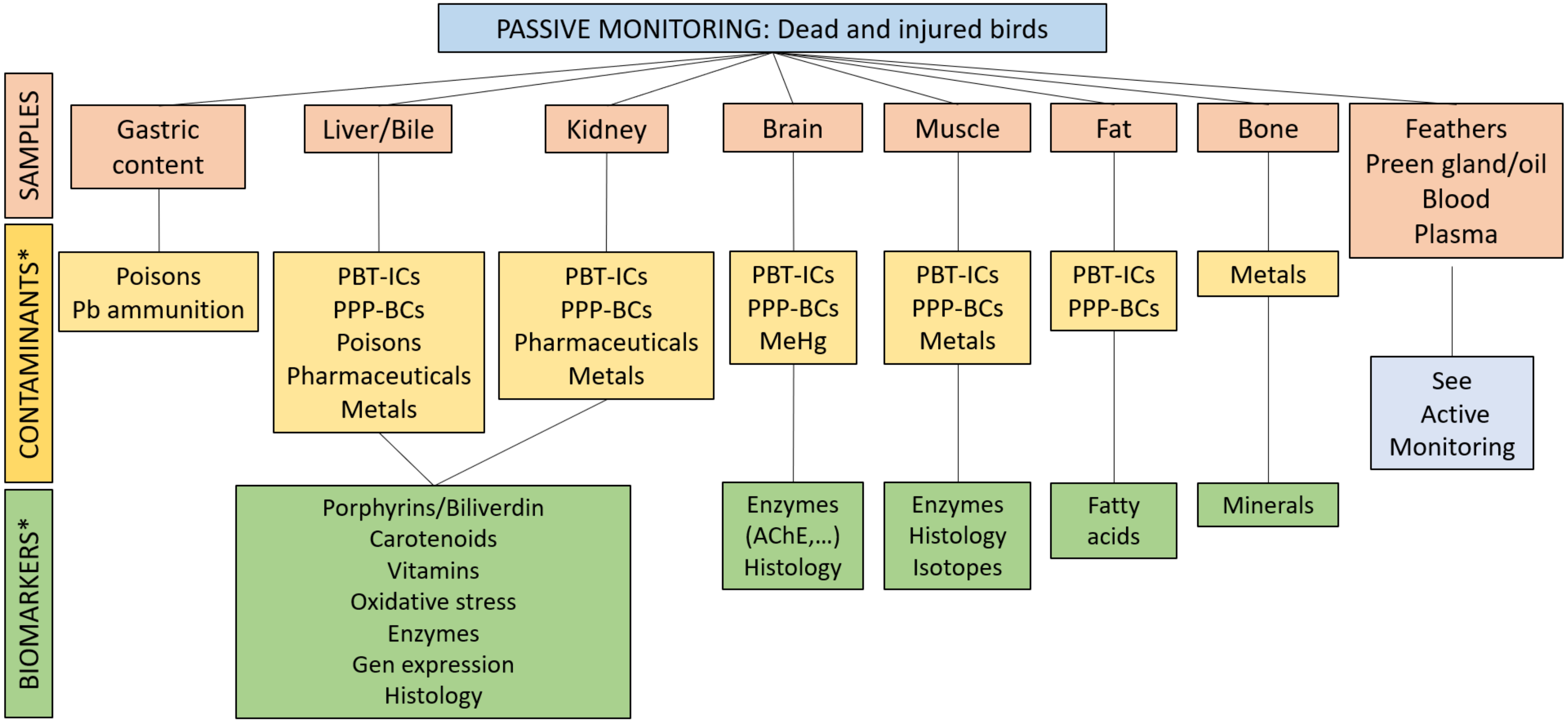

PBT-ICs: persistent bioaccumulative toxicants (chlorinated, brominated) + industrial compounds (fluorinated, PAHs,...)
PPP-BCs: plant protection products and biocides (insecticides, rodenticides, fungicides, herbicides,...)
MeHg: methylmercury / **AChE**: acetylcholinesterase
*Contaminants and biomarkers frequently analyzed in biomonitoring studies are shown. Please see Espín et al. (2016) to see specific merits of each type of sample for contaminant analysis.

See References here
Go back to the main menu here

Schematic protocol for blood/plasma/serum/red blood cells: additional information

Prepare the correct needle and syringe: Take blood samples using a hypodermic needle and a syringe. Change needles between birds. Use the smallest needle possible: for birds < 500 g body weight: 30 to 25-gauge hypodermic needle and a 1-2 ml syringe / > 500 g body weight: 23-gauge hypodermic needle and a 5-10 ml syringe. Volume should be sufficient to ensure suitable analytical limits of detection (see Table 1 and Figure 2a). However, in no circumstances should the collection volume exceed 1% of the body weight.

Use anticoagulants for whole blood/plasma (e.g. heparin: 2-3 drops in a 1.5 ml-tube or heparinized tubes). See Figure 2a. EDTA may be problematic for biochemistry and metals, while heparin may interfere with PCR analysis (more info: Espín et al., 2014).

Collect blood from brachial/jugular/tarsal vein (see Figure 2b): Stimulate the local blood circulation, e.g. by allowing wing flapping before puncturing (for brachial vein collection), use antiseptic at the phlebotomy site and take blood samples puncturing the vein. Press the puncture site with sterile dry cloth or non-woven gauze before pulling the needle from the vein, and keep pressure on the cloth at the puncture site for some minutes to avoid bleeding and haematomas (more info: Espín et al., 2014).

Transfer blood to proper tube: Remove needle before placing the sample in tubes (see Figure 2a). Tubes containing anticoagulants should be adequately filled in order to provide a proper blood-to-anticoagulant ratio.

Transport samples at 4-10 ºC. Avoid direct contact with cold blocks/ice bags and temperatures <4 ºC to avoid haemolysis (see Table 1).

For serum/plasma collection: Use anticoagulants in the tube to obtain plasma, otherwise you will obtain serum. Centrifuge tube as soon as possible (10 minutes, 1600-3000 g), ideally within 6 hours (max. 24 h) after collection; the longer the elapsed time, the higher the risk of clotting and rupture of red blood cells. Plasma/serum/red cells separation is possible on fresh blood only and cannot be done on samples that have been frozen. Use different pipette tips for each sample during plasma/serum separation to avoid cross contamination. Keep all separated fractions (red cells, plasma/serum) in different labelled tubes.

Storage: Keep frozen at -20°C /-80°C /liquid N_2 (depending on the analyte or the studied biomarker; see Table 1). Take advice from the laboratory undertaking the chemical/biochemical analysis for further information about temperature and duration. Specific protocols for biomarkers may exist.

Click here to see Figure 1. What can we measure in each sample type? (a. Active monitoring / b. Passive monitoring)

Click to see video for blood extraction and plasma/serum separation
See References here /Go back to blood protocol here or to plasma/serum protocol here

Figure 2a. Material for blood/plasma/serum/red blood cells sampling.

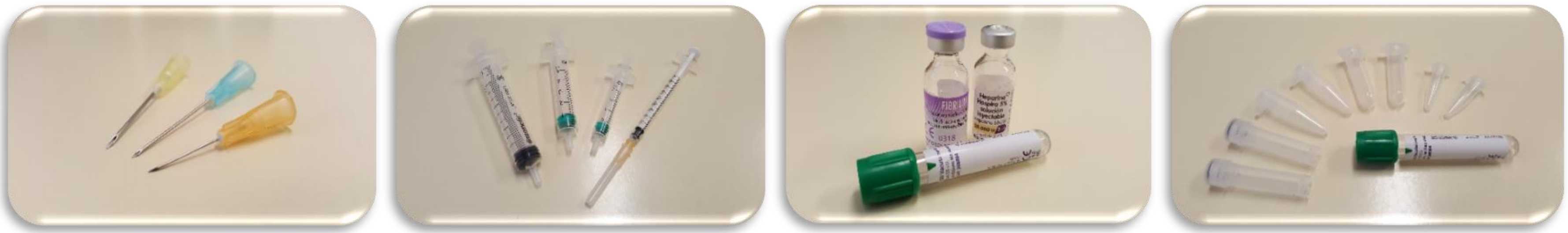

Figure 2b. Blood sampling from brachial, tarsal and jugular veins.

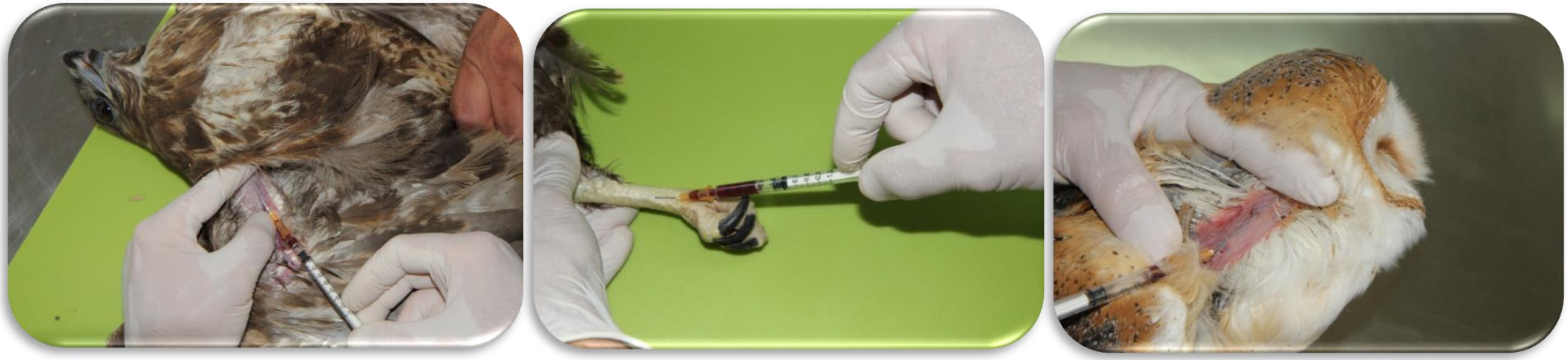

Figures: S. Espín and P. María-Mojica

Go back to blood protocol here or to plasma/serum protocol here

Schematic protocol for deserted and addled eggs: additional information

Collect only deserted eggs or addled eggs from the nest. Be careful about the timing of egg collection to avoid nest abandonment.

Transport eggs in suitable containers (e.g. polypropylene jars, chicken eggs boxes) to avoid breaking (see Figure 3a). Keep cool and process egg as quickly as possible. Use a graphite pencil to write information on both the eggshell and the container. Collect pieces of the eggshells found in the nest and keep them in sealed plastic bags (see Figure 3a), they may be useful for some contaminant analysis.

Take measurements and examine eggs before freezing: Measure length and width, and weigh the egg. Open at the equator of the egg and empty its contents into flasks, weigh and homogenise the content (using clean tools), and keep frozen until analysis (see Table 1). Examine eggs for putrefaction, embryo development (see Figure 3b) and deformities. If an embryo is present, keep frozen for future analyses. Rinse eggshell with tap water to remove all remains of egg contents from the inner surface. Dry eggshells at room temperature to a constant weight, and record the constant eggshell weight. Measure eggshell thickness at equator after drying at room temperature using a calliper (digital if it is possible) to take at least five measurements by the same investigator from the dry shell (see Figure 3a). A micrometer rather than a calliper may provide more comparable measurements.

Transfer egg content and eggshells to proper containers (see Table 1) and **store samples** (homogenized content at -20°C and eggshells at room temperature, see Table 1).

*Click **here** to see Figure 1. What can we measure in each sample type? (a. **Active monitoring** / b. **Passive monitoring**)*

*Click **here** to see video*

*See References **here***

*Go back to egg protocol **here***

Figure 3a. Containers for eggs/eggshells and measurements (for labs).

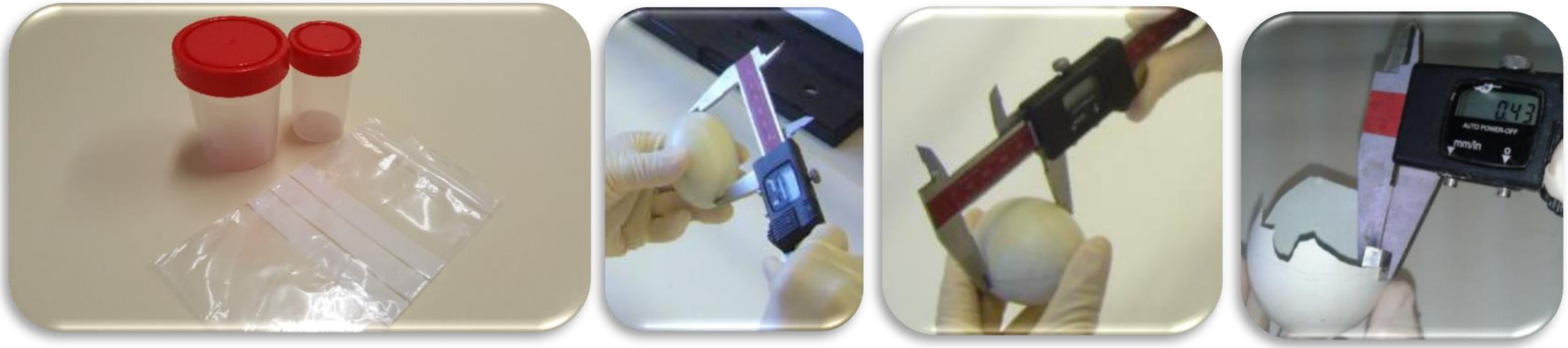

Figure 3b. Embryo development: vascular formation (1), morula or blastocyst (2), embryo in first development stages (3) and very advanced embryo development (4a, b).

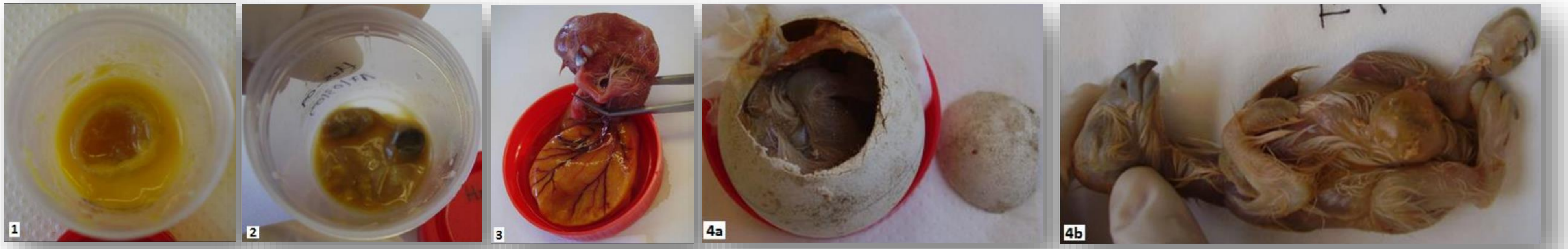

Go back to egg protocol [here](#)

Figures: S. Espín, P. Gómez-Ramírez and L. Ramón

Schematic protocol for feathers: additional information

Collect feathers: Plucked (or cut at the skin) contour body feathers are preferred (see Figure 4). In adult birds, plucking tail or flight feathers should not be collected as it can impair the flight ability of the bird. From dead birds, all feather can be collected. Freshly moulted feathers found in the nest or field can also be collected.

Transport feathers in sealed plastic bags or envelopes (see Figure 4) at ambient temperature or using cold blocks. Before they are stored, feathers that have been plucked from living birds or collected from carcasses should be cleaned of all fresh tissue (blood, muscle) and they should be dried if they are wet. Otherwise, if they are stored at ambient temperature in sealed bags, rotting will occur. Alternatively, freeze the uncleaned feathers in sealed plastic bags. Regarding the sample amount, see Table 1.

Identify and measure feathers: Identify type and number of the feather (left or right). In case of contour feathers, indicate the location on the body. Use the conventional numbering system for primary flight feathers from the inside out. (more info: Espín et al., 2014)

Store feathers: Feathers can be kept at room temperature if stored properly and if any soft tissue or blood residue is removed. Store feathers in plastic sealed bags or envelopes, in darkness, and in a dry place (or use silica) if stored at room temperature. Alternatively, you can freeze the feathers in sealed plastic bags (see Table 1). Container materials should be checked to be free of contamination.

Click here to see video / Click here to see Figure 1. What can we measure in each sample type? (a. Active monitoring / b. Passive monitoring)

Figure 4. Sealed plastic bags and envelopes for feathers and back feathers collection.

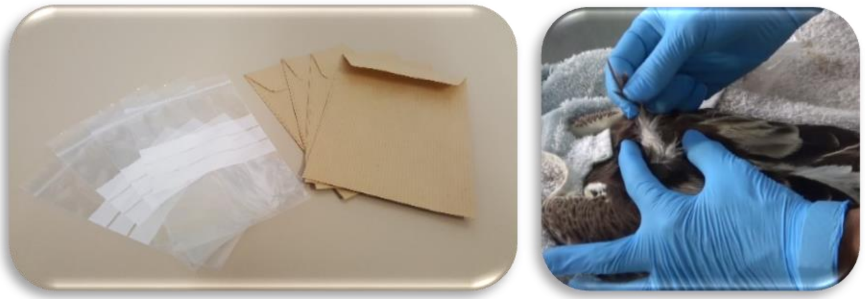

Go back to feathers protocol here / See References here

Figures: S. Espín and P. María-Mojica

Schematic protocol for preen oil: additional information

Press the preen gland softly and collect the expelled oil in a sterile tube in living birds. Two stainless steel spoons (one with a hole) can also be used for oil collection. When sampling from carcasses, the whole preen gland may be removed.

Transport preen oil under cold conditions. Regarding the sample amount, see Table 1.

Store samples at -20°C (see Table 1).

Click here to see Figure 1. What can we measure in each sample type? (a. Active monitoring / b. Passive monitoring)

Go back to preen oil protocol here

Schematic protocol for regurgitate pellets / prey remains: additional information

Regurgitate pellets and prey remains encountered near roosting sites and nests can be collected in **plastic bags or polypropylene flasks**. Collection should not be done during incubation/small nestlings to avoid nest desertion. To collect pellets for contaminant analysis, a first visit to each site is needed in order to remove existing pellets so that subsequent pellets collected will be from a recent period of time (to avoid degradation of some compounds over time). Those that are fresh (having a coating of mucous) can be reserved for prey identification and possible residue analysis. In the second visit, fresh pellets can be collected and placed into individual plastic bags or polypropylene flasks clearly labelled.

Transport under cold conditions using cold blocks.

Stord at -20°C until chemical analysis (see Table 1).

Both old and fresh pellets can also be used to identify prey content.

Click here to see Figure 1. What can we measure in each sample type? (a. Active monitoring / b. Passive monitoring)

Go back to pellets/prey remains protocol here

Schematic protocol for internal tissues/gastric content: additional information

Collect carcasses in sealed plastic bags to avoid dessication and label the bag (with waterproof marker). (More info: Espín et al., 2014)

Transport the carcasses to the lab under cold conditions using cold blocks.

Perform necropsy: Necropsies should be carried out using protocols that avoid both potential exposure of the researcher to zoonotic diseases and chemical contamination of the sample. A proper professional necropsy requires a trained veterinarian (pathologist); however, when the aim is to collect a tissue for contaminant monitoring purposes, trained personnel may collect the samples. Necropsies should be done on fresh carcasses where possible or the carcass should be kept frozen (−20°C) until necropsy. If the carcass is frozen, thaw it overnight. External examination of the carcass is necessary to find possible signs of trauma or evidence of clinical symptoms previous to the death (e.g. haemorrhages, diarrhoea, salivation, etc). The cause of death should be determined if possible with the help of an experienced pathologist. Body/nutritive condition can be estimated as a relative score using the criteria in Figure 5a. During necropsy, record organ weight, lesions/alterations, sex and status of the gonads (developmental stage) (Figure 5b). Take pictures if possible. Take advice from the laboratory undertaking the chemical analysis as to selection of tissues (see Table 1 and Figure 1b). Use suitable dissection material (Figure 5c) and disposable gloves, disinfect instruments and surfaces and clean the material between the organ sampling and between individuals. Regarding sampling of liver, kidney and other internal organs, the whole organ must be taken if possible. In case of muscle sampling, the pectoral muscle is the preferred choice. If sampling tissues are dispersed through parts of the body, such as fat or bone, it is recommended that the tissue is sampled consistently from the same part of the body. Collect all the gastric content. A standardised necropsy protocol should be followed. We provide a necropsy form (Figure 5d) and figures of anatomy of birds (Figure 5e) to facilitate the sampling. (more info: Espín et al., 2014)

Transfer organs/samples to separate containers/sealed plastic bags (to avoid freezer burn) (see Table 1 and Figure 5c) and label the containers.

Store the samples at -20°C or -80°C (depending on the analyte or the studied biomarker; see Table 1). Take advice from the laboratory undertaking the chemical analysis for further information.

Click here to see Figure 1. What can we measure in each sample type? (a. Active monitoring / b. Passive monitoring)

Click here to see video / *Go back to internal tissues protocol here* / *See References here*

Figure 5a. Body condition index (scored according to a four-point scale, from van Franeker, 1983, 2004) and nutritive condition.

Body condition index

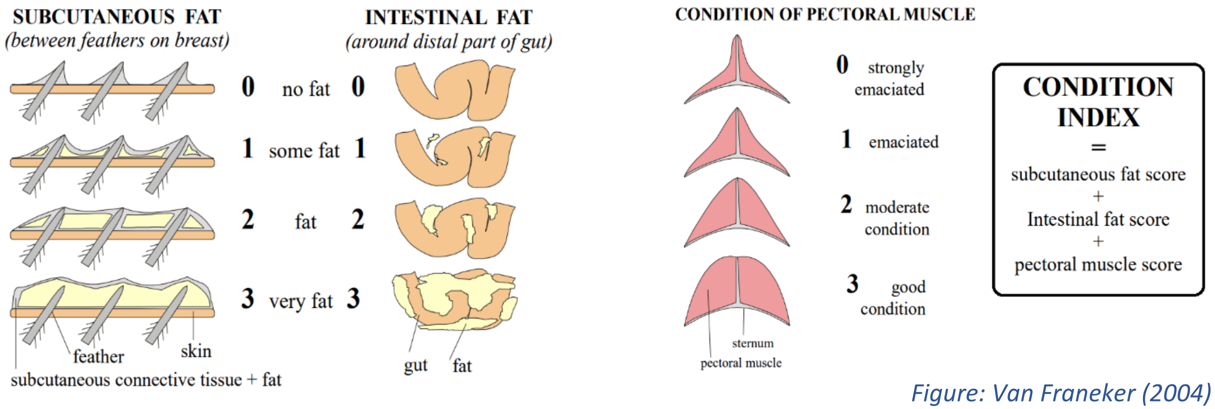

Figure: Van Franeker (2004)

Note: The shape of the breast muscle generally shows active flight behavior. Nestlings often have a bilaterally concave shape, but the birds have a good nutritive condition. Therefore, this method should be used in adult individuals.

Nutritive condition

A method to assess the nutritive condition of a raptor is to measure the subcutaneous fat between the skin and the belly muscles caudal of the sternum, to measure the body fat between the belly muscles and the gizzard/gut at the caudal margin of the sternum and measure the width of the coronary fat.

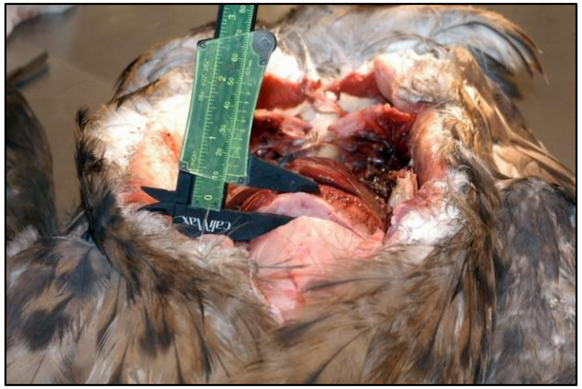

Measuring body fat tissue between the belly muscles and the gizzard. The sternum has already been removed (Figure: O. Krone).

Categorisation of the nutritional condition (fat tissue: X = existing, 0 = not existing)

	Fat tissue		
	**Fat in body cavity**	**Subcutaneous fat**	**Coronary fat**
**Very good**	X	X	X
**Good**	X	X	X
**Moderate**	0	X	X
**Poor**	0	0	X
**Very poor**	0	0	0

Please note that the coronary fat tissue often has a slushy appearance in birds with poor and very poor nutritional condition. In addition, birds in poor and very poor nutritional condition also metabolise proteins including internal organs and muscle tissue.

See References here
Go back to internal tissues protocol here

Figure 5b. Gonads (ovary and testis) of Eurasian Eagle owl.

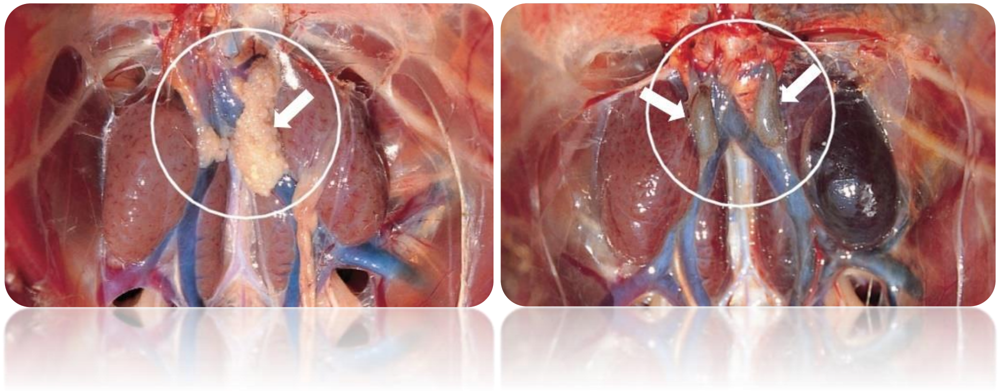

Figure 5c. Necropsy material and containers.

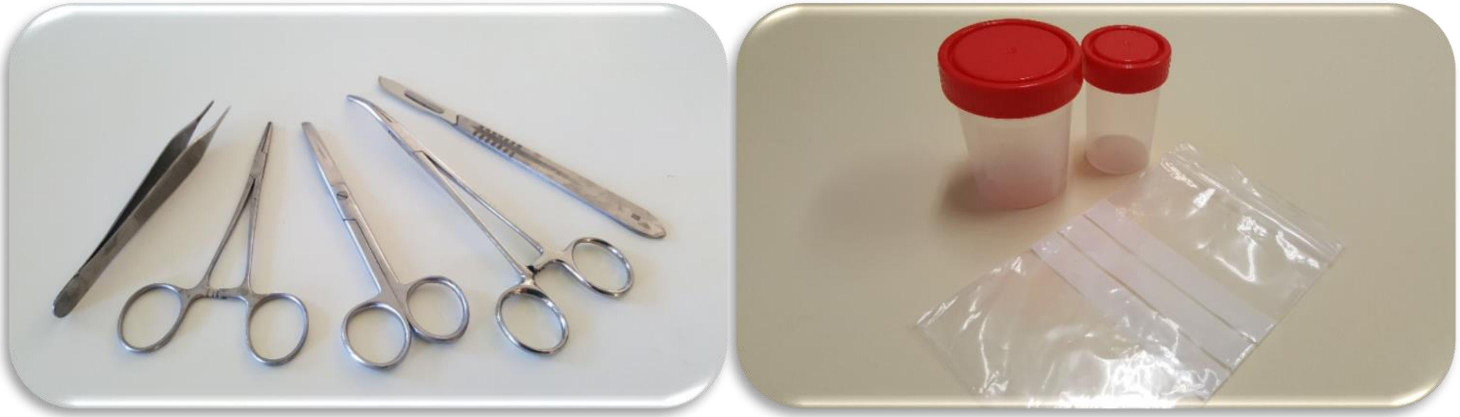

Go back to internal tissues protocol here

Figures: S. Espín and P. María-Mojica

Figure 5d. Example of necropsy form.

**NECROPSY FORM**					
**Carcass information**					
**Species**			**Identification code**		
**Age**			**Sex**		
**Wing length**		**Tarsus length**		**Tarsus width**	
**Head length**		**Bill length**		**Body weight**	
**Location of the carcass**					
**Date collected**					
**Circumstances in which the carcass was found**					
**Collector/Finder (contact information)**					
**Necropsy information**					
**Thawing**	**Date and time**				
**Necropsy**	**Date and time**		**Person in charge**		
**DESCRIPTION OF ALTERATIONS**					
**Status of decomposition**					
**External examination**					
**Cause of death**					
**Body condition**					
**Head and neck**	**Eyes**		**Ears**		
	**Oral cavity**		**Nares**		
	**Brain (weight)**				
	**Observations (lesions/alterations)**				
**Thoracic cavity**	**Pectoral muscle**		**Subcutaneous fat**		
	**Heart (weight)**				
	**Lungs (weight)**		**Trachea**		
	**Observations (lesions/alterations)**				
**Abdominal cavity**	**Liver (weight)**			**Abdominal fat**	
	**Kidney (weight)**				
	**Esophagus**			**Crop**	
	**Intestine**			**Cloaca**	
	**Observations (lesions/alterations)**				
**Gonads**	**Developmental stage**				
	**Observations (lesions/alterations)**				
**SAMPLES COLLECTED FOR CONTAMINANT MONITORING**					
Liver	Kidney	Bone	Feathers	Subcutaneous fat	
Abdominal fat	Brain	Pectoral muscle	Preen oil	Crop content	
Other					

Go back to internal tissues protocol here

Figure: S. Espín

Figure 5e. Anatomy of raptors.

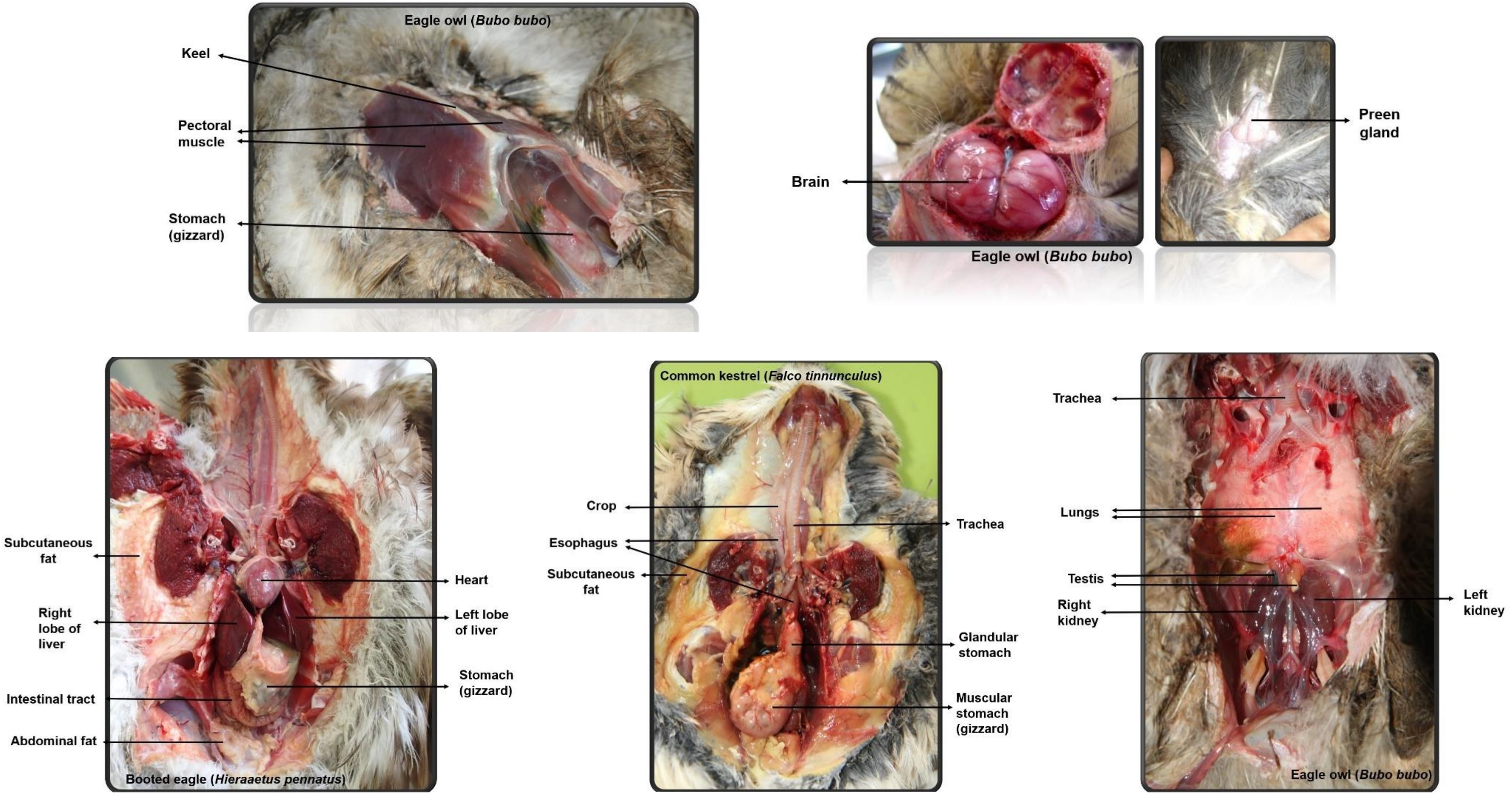

Go back to internal tissues protocol [*here*]

Figures: P. María-Mojica and P. Gómez-Ramírez

References

An extended Sampling and Contaminant Monitoring Protocol For Raptors is freely available on the EURAPMON website (http://www.eurapmon.net)

- Espín S, García-Fernández AJ, Herzke D, Shore RF, van Hattum B, Martínez-López E, Coeurdassier M, Eulaers I, Fritsch C, Gómez-Ramírez P, Jaspers VLB, Krone O, Duke G, Helander B, Mateo R, Movalli P, Sonne C, van den Brink NW. 2014. Sampling and Contaminant Monitoring Protocol for Raptors. Research Networking Programme-EURAPMON (Research and monitoring for and with raptors in Europe). www.eurapmon.net
- Espín et al. 2016. Tracking pan-continental trends in environmental contamination using sentinel raptors-what types of samples should we use? Ecotoxicology 25(4): 777-801.
- MacLachlan, D., Lunn, D. 2003. Methamidophos (100). First draft prepared by D. MacLachlan, Australian Government Department of Agriculture, Fisheries and Forestry, Australia, and D. Lunn, New Zealand Food Safety Authority, New Zealand. Available online: http://www.fao.org/fileadmin/templates/agphome/documents/Pests_Pesticides/JMPR/Evaluation03/methamidophos_2003.pdf
- O'Brien, J., Campbell, N., Conaghan, T. 1981. Effect of cooking and cold storage on biologically active antibiotic residues in meat. J Hyg 87, 511-523.
- VanderKop, P.A., MacNeil, J.D., Patterson, J.R. 1989. Tissue-dependent degradation of monensin residues in chicken tissues with prolonged freezer storage. J Vet Diagn Invest 1, 176-177.
- Van Franeker, J.A. 1983. Inwendig onderzoek aan zeevogels. Nieuwsbr. NSO 4(4/5), pp 144-167.
- Van Franeker, J.A. 2004. Save the North Sea Fulmar-Litter-EcoQO Manual Part 1: Collection and Dissection Procedures. Alterra-rapport 672, Alterra Groen Ruimte, Texel.
- Vindenes et al. 2008. Bromadiolone Poisoning: LC–MS Method and Pharmacokinetic Data. Journal of Forensic Sciences. Volume 53, Issue 4.

Go back to the main menu [here]
